# Beyond Neighborhood Food Environments: Distance Traveled to Food Establishments in 5 US Cities, 2009–2011

**DOI:** 10.5888/pcd12.150065

**Published:** 2015-08-06

**Authors:** Jodi L. Liu, Bing Han, Deborah A. Cohen

**Affiliations:** Author Affiliations: Bing Han, Deborah A. Cohen, RAND Corporation, Santa Monica, California.

## Abstract

**Introduction:**

Accurate conceptualizations of neighborhood environments are important in the design of policies and programs aiming to improve access to healthy food. Neighborhood environments are often defined by administrative units or buffers around points of interest. An individual may eat and shop for food within or outside these areas, which may not reflect accessibility of food establishments. This article examines the relevance of different definitions of food environments.

**Methods:**

We collected data on trips to food establishments using a 1-week food and travel diary and global positioning system devices. Spatial-temporal clustering methods were applied to identify homes and food establishments visited by study participants.

**Results:**

We identified 513 visits to food establishments (sit-down restaurants, fast-food/convenience stores, malls or stores, groceries/supermarkets) by 135 participants in 5 US cities. The average distance between the food establishments and homes was 2.6 miles (standard deviation, 3.7 miles). Only 34% of the visited food establishments were within participants’ neighborhood census tract. Buffers of 1 or 2 miles around the home covered 55% to 65% of visited food establishments. There was a significant difference in the mean distances to food establishments types (*P* = .008). On average, participants traveled the longest distances to restaurants and the shortest distances to groceries/supermarkets.

**Conclusion:**

Many definitions of the neighborhood food environment are misaligned with individual travel patterns, which may help explain the mixed findings in studies of neighborhood food environments. Neighborhood environments defined by actual travel activity may provide more insight on how the food environment influences dietary and food shopping choices.

## Introduction

Research on the influence of neighborhood exposures on dietary behaviors has driven the implementation of programs and policies aimed to improve access to healthy foods (1). The Healthy Food Financing Initiative, for example, is a $400 million annual investment to increase grocery stores in food deserts (2). Although measurements of food access depend on how neighborhood environments are conceptualized, definitions of neighborhoods have varied (3,4). Circular buffers around home, work, or school (5–9) and administrative units such as census tracts (10–13) are commonly used to define neighborhoods, often in studies of food deserts. The US Department of Agriculture (USDA) defines food deserts as low-income census tracts with low access to healthy food, meaning residence more than 1 mile away from a grocery store or supermarket in urban areas (or 10 or 20 miles in rural areas) (1,14). Although a 1-mile radius is often associated with urban food deserts, individuals frequently travel farther to purchase food, with distances averaging 3 miles or more from home (7,15–17). Analyses of the relationships between diet or weight and the neighborhood food environment have mixed results (4,5,18); some findings suggest a relationship (6,12,13), and others suggest no significant relationship (8,9). Mixed findings may result partly because neighborhood definitions vary and because not all food consumption and shopping occur in neighborhoods. Moreover, living in neighborhoods with limited access to healthy foods may not matter if access to transportation allows residents to shop elsewhere.

Given that individuals often travel farther than 1 mile to visit food establishments, researchers have challenged the use of neighborhoods defined by administrative units or buffers (7,8,11,18–20). The objective of this study was to examine the relevance of different definitions of neighborhood food environments by quantifying individuals’ travel beyond commonly defined neighborhoods for food shopping or eating away from home.

## Methods

The study sample consisted of 241 adults in 5 US cities: Los Angeles, California; Chapel Hill, North Carolina; Albuquerque, New Mexico; Columbus, Ohio; and Philadelphia, Pennsylvania. Study participants were recruited from parks and surrounding neighborhoods from May 2009 through April 2011 to participate in an observational study of physical activity and food and travel behavior (21,22). Participants were recruited from 6 (in Chapel Hill, Albuquerque, Columbus, and Philadelphia) or 7 (in Los Angeles) parks in each city (31 parks total). For about half of the parks, the neighborhoods within 0.5 mile around the parks had a higher percentage of households in poverty than the local city or county poverty rate. In the other half of parks, the percentage of households in poverty was lower than the local poverty rate. A monetary incentive of $200 to $225 was provided to each participant at the completion of data collection. Data collection was approved by the institutional review boards at RAND, the University of North Carolina-Chapel Hill, the Pacific Institute for Research and Evaluation, Ohio State University, and the University of Pennsylvania. Written informed consent was provided by all participants.

Participants wore global positioning system (GPS) monitors (Qstarz BT-Q1000X, Qstarz International Co, Ltd) and ActiGraph accelerometers (GTIM, ActiGraph, LLC) for 3 weeks. Location data were collected in 1-minute intervals. Participants were instructed to wear the devices beginning when they woke up each morning to when they went to sleep at night. During the third week of the study, participants also completed a food and travel diary questionnaire on a personal digital assistant (Palm Z22, Palm Inc). Participants were instructed to complete an entry for every trip they took. The diary included questions on the type of place, transportation mode (how they arrived), type of food and beverages consumed, and the type of eating place. The response options for the type of place were home, work, school, sit-down restaurant, fast-food/convenience store, grocery/supermarket, mall or store, someone else’s home, park, community activity facility, place of worship, and other. Participants were given the option to write in a response under other. In this article, sit-down restaurants, fast food/convenience stores, grocery/supermarkets, and malls or stores are considered food establishments that are part of the food environment. Analyses of the accelerometer data, type of food responses, and within place characteristics are reported elsewhere (22,23).

### Spatial-temporal analysis

We conducted a spatial-temporal cluster analysis to identify the locations of destinations of interest corresponding to home and food establishments recorded in the diary. Analyses were mainly conducted in R version 3.1.0 (R Foundation for Statistical Computing).

First, we applied a hierarchical spatial cluster analysis to identify spatial clusters based on GPS latitude and longitude coordinates for each person-day. The average distance between points within each cluster was used to define spatial clusters. All spatial clusters were at least 100 meters apart. Spatial clusters where people stayed for 5 or more minutes were further evaluated as possible destinations of interest in the temporal analysis, while smaller clusters (such as singletons of 1 minute) were deemed as points during travel and omitted. Home locations for each person-day were identified by the spatial clusters corresponding to the arrival time of trips back to home reported in the diary.

Next, we conducted a temporal analysis to match spatial clusters to food establishments. Diary records indicating a trip to a food establishment were matched to spatial clusters based on time. When an exact match was not found, we searched within a 40-minute window from the reported time in the diary and assigned the spatial cluster nearest in time as the match. The rationale for the search within a time window is that the diary time was self-reported and subject to recall error. Diary records with no matched or multiple matched clusters were excluded from the analysis. In a trip from home to a food establishment, if the recalled departure time was earlier than the actual departure time, the spatial cluster of the home location could be mismatched to the diary record, resulting in a zero distance between home and the food establishment. We examined all matched trips to a food establishment within 200 meters from home. If a second nonhome spatial cluster within 30 minutes before and after the diary time was identified, the second cluster was assigned as the food establishment location.

### Statistical analysis

Descriptive statistics were calculated to summarize the trips to food establishments and the distance from home, by the type of food establishments. Euclidean distances were calculated between food establishment locations and participants’ homes. A 1-way analysis of variance was used to determine whether the distances were different by type of establishment. Two broad types of neighborhood food environment definitions were considered: circular buffers around homes and census tracts with polygon-shaped buffers. Buffer radii were set at 0.5, 1, 2, and 5 miles around homes. Buffers of 0.5 to 2 miles are commonly used to define neighborhood environments. Although a 5-mile buffer covers a large area that may be beyond what is typically considered a neighborhood, we included buffers with 5-mile radii for sensitivity analysis. In addition to census tracts to delineate a neighborhood, environments defined by census tracts plus buffers of 0.5, 1, and 2 miles from census tract boundaries were also assessed. Polygon-shaped buffers around the census tracts were generated using ArcGIS (Esri). The coverage of the neighborhood food environment definitions was calculated as the proportion of food establishments visited by participants that were located within the defined food environment area. Confidence intervals (CIs) for the proportions were calculated using a *z*-test statistic.

## Results

Of the 241 park study participants, 135 (56%) participants reported travel to a food establishment during the 1-week diary period and had a home location and at least 1 food establishment location identified by the spatial-temporal clustering algorithm. Participants in the analysis sample had a mean age of 41 years (standard deviation [SD], 15 y). The sample was 57% female, 53% non-Hispanic white, 16% non-Hispanic black, and 18% Hispanic (Table 1). The education level of participants ranged from high school diploma or less to postgraduate. Average body mass index was 27.6 kg/m^2^ (SD, 5.9 kg/m^2^).

**Table 1:** Participant Characteristics (N = 135), Distance Traveled to Food Establishments in 5 US Cities, 2009–2011

	No. (%)
**Age, y**	
18–35	64 (47)
36–59	46 (34)
60–85	24 (18)
Missing	1 (1)
**Sex**	
Female	77 (57)
Male	57 (42)
Missing	1 (1)
**Race/ethnicity**	
Non-Hispanic white	72 (53)
Non-Hispanic black	21 (16)
Hispanic	24 (18)
Other	17 (13)
Missing	1 (1)
**Education**	
Some high school or GED	19 (14)
Some college or vocational school	36 (27)
College	50 (37)
Postgraduate	29 (21)
Missing	1 (1)
**Body mass index, kg/m^2^**	
18–<25	50 (37)
25–<30	45 (33)
≥30	39 (29)
Missing	1 (1)
**City**	
Los Angeles, California	40 (30)
Chapel Hill, North Carolina	38 (28)
Albuquerque, New Mexico	29 (21)
Columbus, Ohio	23 (17)
Philadelphia, Pennsylvania	5 (4)

Abbreviation: GED, general educational development.

We identified 513 visits to food establishments (Table 2). Of these, 43 mismatches within 200 meters from home were reassigned to the next nearest spatial cluster. We excluded 79 mismatches for which multiple spatial clusters were identified and we could not reliably determine the correct cluster. Matched food establishments greater than 20 miles away from the home were also excluded, because the trips were well beyond what could be reasonably considered part of an individual’s routine travel activity in their neighborhood. We found an average of 2 visits to food establishments per participant during the 1-week diary period. 

**Table 2:** Characteristics of Food Establishments Visited by Participants (N = 135), by Type of Food Establishment, 5 US Cities, 2009–2011

Characteristic	All Food Establishments	Sit-Down Restaurant	Fast-Food/ Convenience Store	Mall or Store	Grocery/ Supermarket
No. of people with ≥1 visit	135	89	64	54	51
Number of visits	513	173	122	111	107
**Visits per person**					
Mean (SD)	2.0 (1.3)	1.3 (1.4)	0.9 (1.3)	0.8 (1.3)	0.8 (1.3)
Median (IQR)	2 (1–2)	1 (0–2)	0 (0–1)	0 (0–1)	0 (0–1)
Range	1–8	1–7	1–6	1–7	1–8
**Distance from home, miles**					
Mean (SD)	2.6 (3.7)	3.3 (4.3)	2.4 (3.7)	2.3 (3.4)	1.9 (2.9)
Median (IQR)	0.7 (0–4.0)	1.4 (0–4.9)	0.6 (0–3.4)	0.7 (0–3.5)	0.4 (0–2.5)
Range	0–19.4	0–18.0	0 –19.5	0–15.9	0–12.3

Abbreviations: SD, standard deviation; IQR, interquartile range.

The average Euclidean distance between a food establishment and home was 2.6 miles (SD, 3.7 miles) (Table 2). Differences in distances from home across the food establishment types were significant (*P* = .008). On average, sit-down restaurants visited by participants were farthest from home (3.3 miles), and grocery stores visited by participants were closest to home (1.9 miles). The median distance to sit-down restaurants was 1.4 miles, compared with 0.6 miles to fast food/convenience stores, 0.7 miles to malls or stores, and 0.4 miles to grocery/supermarkets. The distributions of the distances between matched food establishment locations and participants’ homes were highly skewed for every type of food establishment (Figure). Although many food establishments were close to the home, the distributions had a long tail beyond 5 miles from home. Distances of trips to grocery/supermarkets appeared to have the smallest spread, and distances of trips to sit-down restaurants were the most widely distributed.

**Figure:**
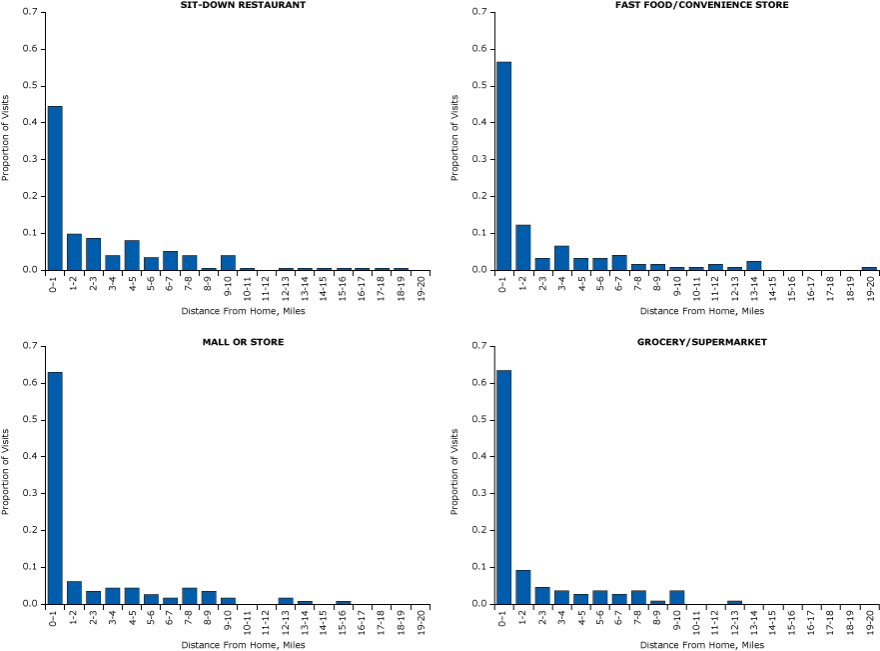
Histograms of distances between home and food establishments, by type of food establishment. Distance traveled to food establishments in 5 US cities, 2009–2011. **Table:** 

Distance From Home, Miles	Type of Establishment, Proportion of Visits			
	Sit-Down Restaurant	Fast-Food/Convenience Store	Mall or Store	Grocery/Supermarket
0–1	0.45	0.57	0.63	0.64
1–2	0.10	0.12	0.06	0.09
2–3	0.09	0.03	0.04	0.05
3–4	0.04	0.07	0.05	0.04
4–5	0.08	0.03	0.05	0.03
5–6	0.03	0.03	0.03	0.04
6–7	0.05	0.04	0.02	0.03
7–8	0.04	0.02	0.05	0.04
8–9	0.01	0.02	0.04	0.01
9–10	0.04	0.01	0.02	0.04
10–11	0.01	0.01	0	0
11–12	0	0.02	0	0
12–13	0.02	0.01	0.02	0.01
13–14	0.01	0.02	0.01	0
14–15	0.01	0	0	0
15–16	0.01	0	0.01	0
16–17	0.01	0	0	0
17–18	0.01	0	0	0
18–19	0.01	0	0	0
19–20	0	0.01	0	0

Table 3 shows the coverage of food establishments by several commonly used neighborhood food environment definitions, on the basis of circular buffers around homes and census tract boundaries. Neighborhood definitions using small circular buffers of 0.5- and 1-mile radii around homes covered between 37% and 64% of the different food establishment types, where 37% (95% CI, 30%–45%) of sit-down restaurants were within 0.5 miles from homes and 64% (95% CI, 54%–73%) of grocery/supermarkets were within 1 mile from homes. A 2-mile buffer around the home covered more, from 54% of sit-down restaurants to 73% of grocery/supermarkets. Census tracts had an overall coverage of 34% of food establishments (25% of sit-down restaurants to 41% of grocery/supermarkets). Polygon-shaped buffers around census tracts improved coverage, with 2-mile buffers around census tracts covering 56% of sit-down restaurants and 76% of grocery/supermarkets. The 2-mile circular buffers around homes are not directly comparable with 2-mile polygon-shaped buffers around census tracts, because the latter can be substantially larger than the former. The median polygon-shaped buffer of 2 miles around census tracts was 21.5 square miles. By contrast, a circular buffer with a 2-mile radius covers 12.6 square miles. The largest circular buffer, with a 5-mile radius, is rarely used to define neighborhoods and covers 78.5 square miles. This expansive area covered 80% of food establishments, with 75% of sit-down restaurants and 84% of grocery/supermarkets visited.

**Table 3:** Coverage of Food Establishments Visited by Participants (N = 135), by Neighborhood Food Environment Definitions and Type of Food Establishment, 5 US Cities, 2009–2011

Spatial Definition of Neighborhood Food Environment	Coverage of Food Establishments, % in Neighborhood (95% CI)				
	All Food Establishments	Sit-Down Restaurant	Fast Food/ Convenience Store	Mall or Store	Grocery/ Supermarket
**Circular buffer radii around homes, miles (square miles)**					
0.5 (0.8)	45 (40–49)	37 (30–45)	48 (39–56)	45 (36–54)	52 (43–62)
1 (3.1)	55 (51–60)	45 (37–52)	57 (48–65)	63 (54–72)	64 (54–73)
2 (12.6)	65 (61–69)	54 (47–61)	69 (61–77)	69 (61–78)	73 (64–81)
5 (78.5)	80 (77–84)	75 (69–82)	82 (75–89)	82 (75–89)	84 (77–91)
**Census tract (CT) boundaries^a^ (median, square miles)**					
CT (0.8)	34 (29–38)	25 (19–32)	39 (30–47)	33 (25–42)	41 (32–50)
CT + 0.5-mile buffer (3.7)	51 (47–56)	38 (30–45)	57 (48–65)	56 (47–65)	63 (53–72)
CT + 1-mile buffer (8.1)	59 (54–63)	47 (40–55)	61 (53–70)	64 (55–73)	68 (59–77)
CT + 2-mile buffer (21.5)	67 (63–71)	56 (49–63)	70 (62–79)	72 (64–80)	76 (68–84)

Abbreviation: CI, confidence interval.

a Median areas of census tracts are based on the census tracts containing the participants’ home.

## Discussion

Substantial national attention and investment has been focused on food deserts as defined by the USDA, even though fewer than 5% of Americans live in such areas (1). Although our sample was not recruited from food deserts, proximity did not appear to substantially limit where participants shopped for food or ate away from home. Given that people often travel beyond neighborhoods for food, focusing on local neighborhoods may be less relevant than other factors affecting healthy eating, such as the ubiquity of unhealthy food in both food retail and nonfood retail stores (24).

In this study, many commonly used definitions for neighborhood food environments had limited coverage of food establishments visited by participants. Circular buffers of 1- and 2-mile radii around homes are frequently used to define neighborhoods and covered approximately 55% and 65% of the visited food establishments. Census tracts with a 0.5- or 1-mile polygon-shaped buffer around the census tract boundaries covered a similar proportion of the activity. The smallest circular buffer around homes (0.5 miles) and census tracts covered less than half the food establishments visited by participants. The 0.5-mile buffer around a home provided more than 10% greater coverage than census tract boundaries, which typically have similarly sized neighborhood areas of 0.8 square miles. Therefore, circular buffers around the home may provide slightly better coverage than polygon-shaped neighborhoods defined by census tracts, provided that the areas are of similar size. Census tracts are convenient administrative units that allow use of other community and demographic information collected in the census; however, census tracts are crude approximations of neighborhoods. There will always be homes near the edges of census tract boundaries, and residents may very frequently travel outside of their home census tract. Moreover, the size of census tracts is heterogeneous; sparsely populated areas are part of very large census tracts. In this analysis sample, the 25th and 75th percentiles of census tract areas were 0.4 and 2.0 square miles; the largest 5% of census tracts ranged from 8.9 to 1,792 square miles. Another administrative unit that has been used to define neighborhoods is census block groups (4), which are the smallest administrative unit used by the census bureau. However, given that the census tracts in this analysis sample already had less than adequate coverage of food establishments visited by participants, census block groups were not explored as a neighborhood definition in this study. A very large neighborhood definition (eg, a 5-mile buffer around the home) can cover many food establishments but also covers many irrelevant locations. Neighborhood definitions based on large areas are less likely to be meaningful to an individual’s travel activity. There is an inherent trade-off between defining large areas to cover all travel activity and defining a meaningful area that captures commonly traveled places.

The mixed findings in the literature on the effects of food environments may be partly explained by the lack of accuracy in the definitions of neighborhood food environment. For all definitions considered in this article, a substantial proportion of individuals’ actual trips to food establishments would have been missed, and many irrelevant locations in the buffers were included. This misalignment introduces sizeable measurement errors to variables describing individuals’ food environment characteristics. Estimated effects would be systematically biased because of the “errors-in-predictors” problem (25) (ie, regression estimates are biased if the predictors are measured with errors). The biases are usually negative (called *regression attenuation* in the literature) but may also be positive in multiple regressions (26). Although there are advanced methods to account for measurement errors in predictors, they were seldom applied in the studies of neighborhood food environments.

All the neighborhood definitions considered in this study were determined by home locations. These mechanical and rigid definitions fail to capture the heterogeneity among individuals’ actual travel activity. Alternatively, researchers have proposed individualized measures of food environment based on travel areas that allow for more insight on how the food environment influences dietary and food shopping choices (27–30). The spatial-temporal analysis of this article is one approach to objectively analyze individual travel activity. Conceivably, individualized measures are more expensive to collect and more difficult to analyze but can offer more accurate measurements of exposure to food environments.

This study has several limitations. First, the sample size is small. Participants were recruited from parks and nearby areas, and there may be selection bias. Although participants were recruited from 5 major cities, the sample may not be nationally representative of all major cities. The results cannot be generalized to smaller cities and rural areas. However, it is likely that the neighborhood food environments in less urbanized areas are even larger than in major cities. Other limitations are related to data collection. Self-reported diaries are subject to underreporting and recall bias, and there may have been misclassifications in matching self-reported diary times and GPS time points.

Neighborhood definitions of the food environment are crude measures that are often misaligned with individual travel patterns. Approximately one-third to two-thirds of food establishments visited by participants were within commonly used neighborhood food environment definitions. Comparing types of food establishments, sit-down restaurants visited by study participants were more often farther from home and located outside neighborhoods, and grocery stores and supermarkets were more often within the defined neighborhoods. Our use of GPS to monitor individual travel activity allowed for an objective measure of environmental exposures. Measures of the food environment based on actual travel activity may provide more insight on how the food environment influences dietary and food shopping choices.
